# Supplementary material for: From interaction networks to interfaces, scanning intrinsically disordered regions using AlphaFold2
Source: Nat Commun. 2024 Jan 18;15:597. doi: 10.1038/s41467-023-44288-7 (PMC10796318; doi:10.1038/s41467-023-44288-7)
Supplement: Supplementary file 1 — Supplementary Information [file 41467_2023_44288_MOESM1_ESM.pdf]

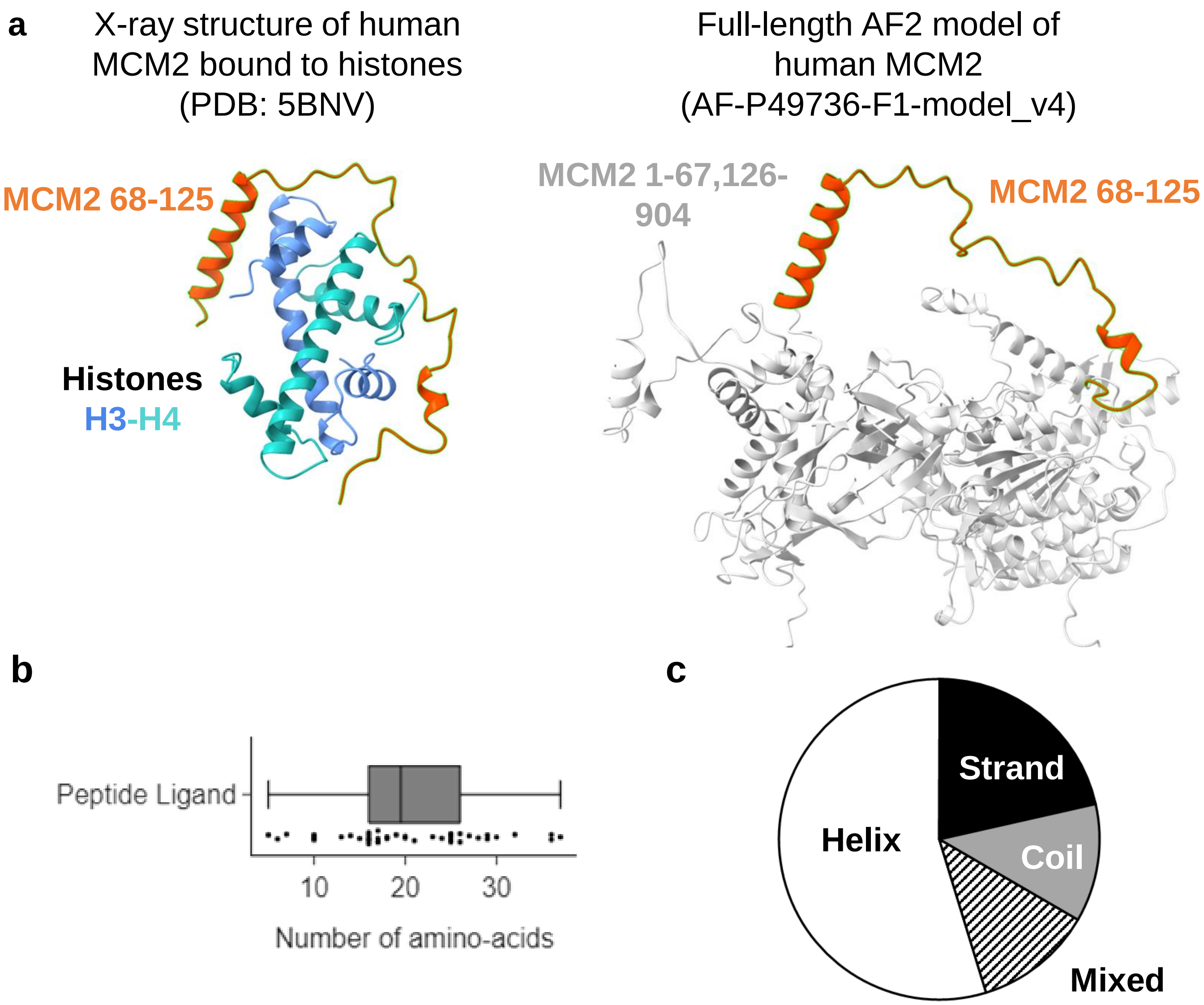

Supplementary Figure 1. Benchmark dataset construction.
(a) (Left) Structure of the intrinsically disordered region (IDR) of human MCM2 (orange) with boundaries [68-125] as experimentally resolved in complex with histones H3 (blue) and H4 (cyan) (PDB: 5BNV) compared (right) to the full AlphaFold model of MCM2 extracted from the AlphaFold database and colored orange in the same boundaries [68-125]. No other structure of this region is available in the PDB. The similarity between the local structures in this stretch highlights that the conformations in the model may be strongly inspired from experimental structures used in the training process of AlphaFold. **(b)** Boxplots representing the size distribution of the 42 peptides in the benchmark data set. **(c)** Classification of the different types of secondary structures observed in the ligands of the 42 complexes in the benchmark database. Four categories were distinguished, peptide structures comprising only helical secondary structure (Helix) or only strand(s) (Strand), peptides binding in the absence of a canonical secondary structure (Coil), and those adopting more complex combinations of the three elements mentioned above (Mixed).

a

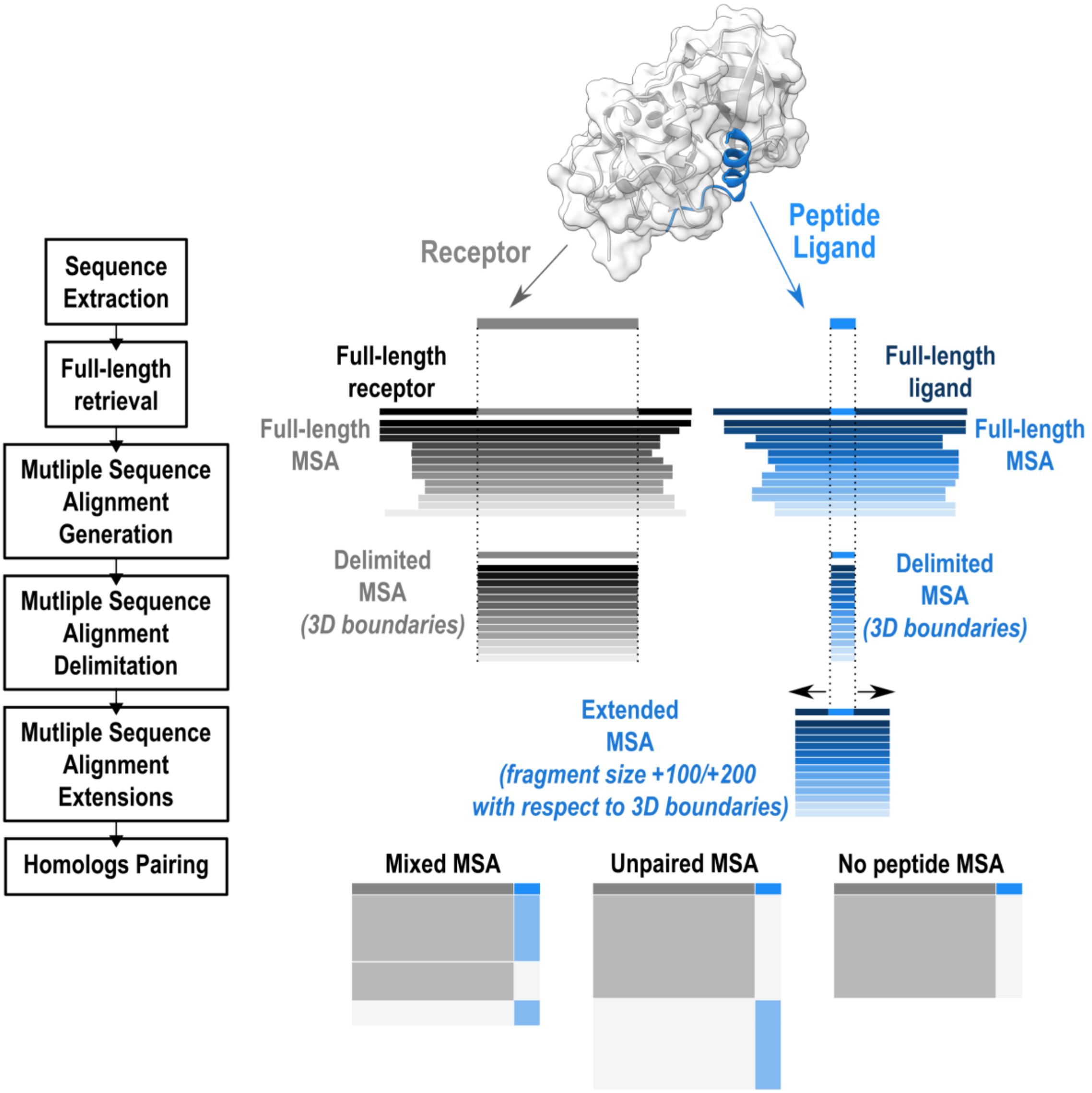

b

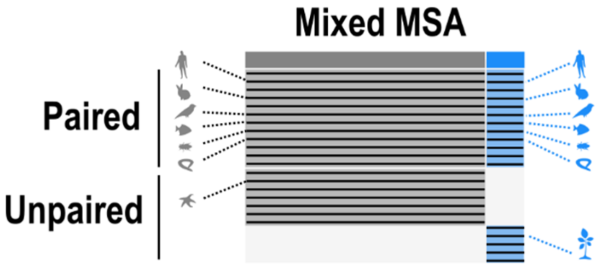

Supplementary Figure 2. Pipeline for the construction of different multiple sequence alignments used in this study.
(a) General pipeline description with schematic representation of the different steps on the right. First, the full-length sequence is retrieved and used for MSA generation with MMseqs (see Methods). Then, delimited alignments are extracted by retrieving only columns corresponding to positions present in the 3D structure. For some predictions, peptide alignments are extended by up to 100 or 200 residue positions. Finally, three modes are used to combine evolutionary information of the receptor and peptide: a mixed alignment in which as many partner sequences as possible are paired while sequences with a single partner homolog present in a species are added as unpaired, a fully unpaired alignment in which no sequences are paired, and a mode with no evolutionary information for the peptide. **(b)** Illustration of the composition of a mixed co-alignment combining paired and unpaired alignments blocks.

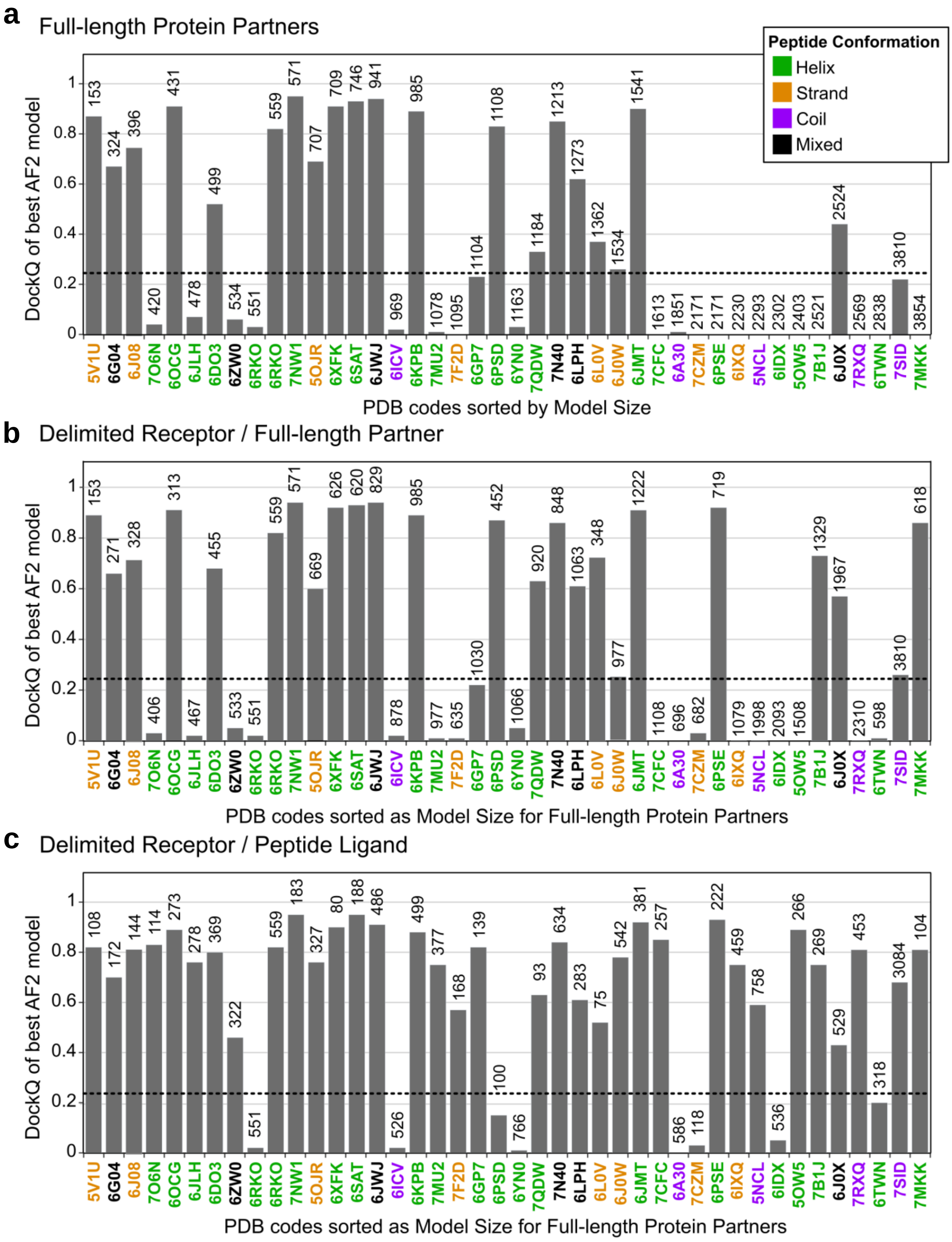

Supplementary Figure 3. Prediction quality of individual test cases for different prediction modes.

Illustrations of the DockQ score of the best predicted model (chosen as the best AF2 combined score) for each of the 42 test cases. Test cases are identified by their PDB code and sorted by the cumulative length of the full-length input sequences (receptor+ligand) in the three panels a-c. The length of each system in each panel is indicated on top of each bar. PDB codes are color-coded according to the peptide bound conformation: helical (green), strand (orange), coil (purple) or a mixture (black). The dashed line indicates a DockQ score of 0.23, which is the threshold for an acceptable prediction as evaluated on protein-protein complexes (see Methods). Three prediction modes are used: **(a)** full-length partners, **(b)** delimited receptor and full-length ligand and **(c)** delimited receptor and ligand. All predictions in this figure are made with mixed alignments.

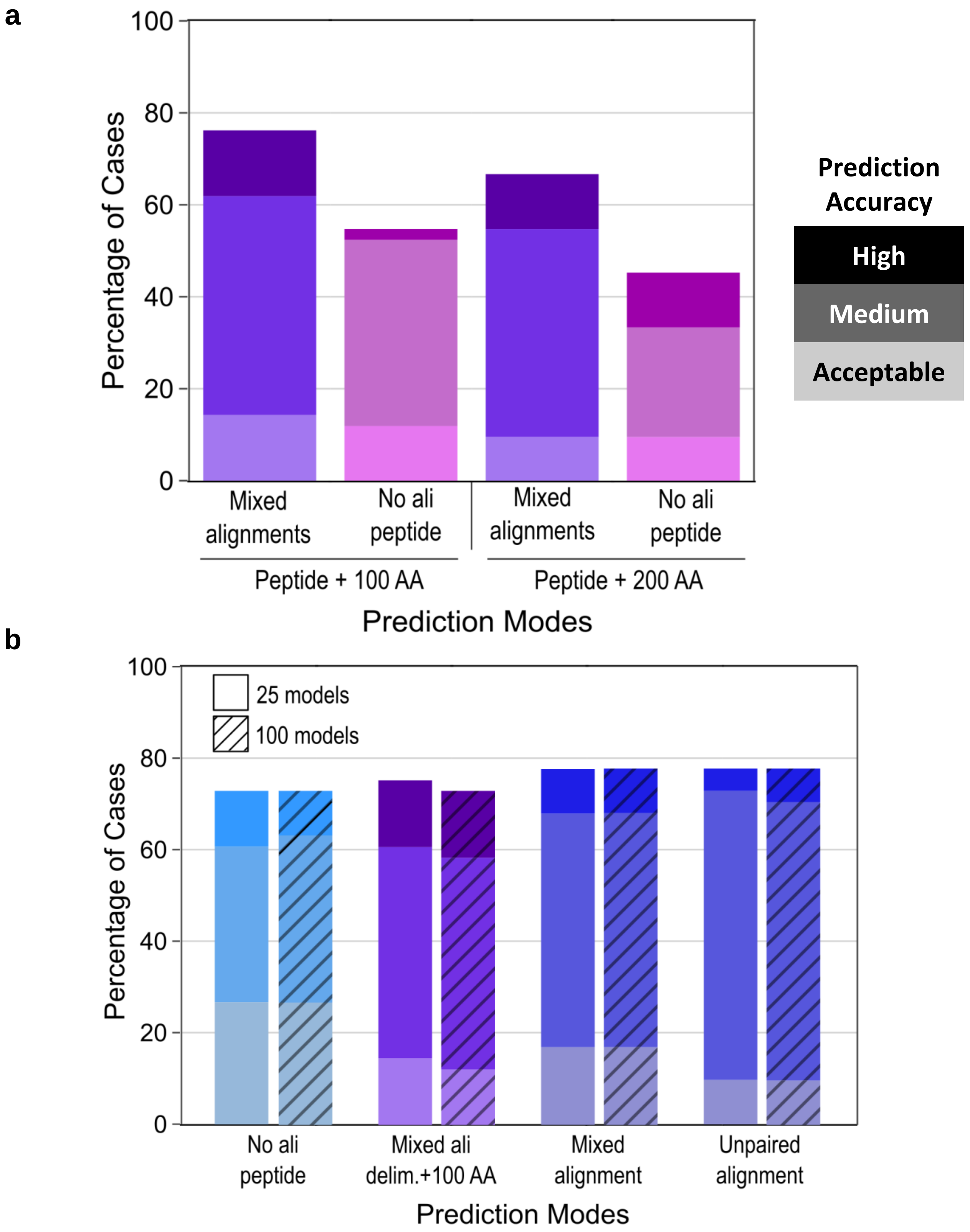

Supplementary Figure 4. AlphaFold2-Multimer success rates on the benchmark dataset using ligands extended by fragments of size 100 and 200.
(a) Success rates as in main Figure 2, calculated for ligands extended by fragments of size 100 (for the two leftmost bars) and 200 (for the two rightmost bars) with either a mixed alignment or no peptide alignment. **(b)** Success rates calculated for four different alignment protocols with the color code as in main Figure 2 when either 25 (plain bars) or 100 (hatched bars) models were generated.

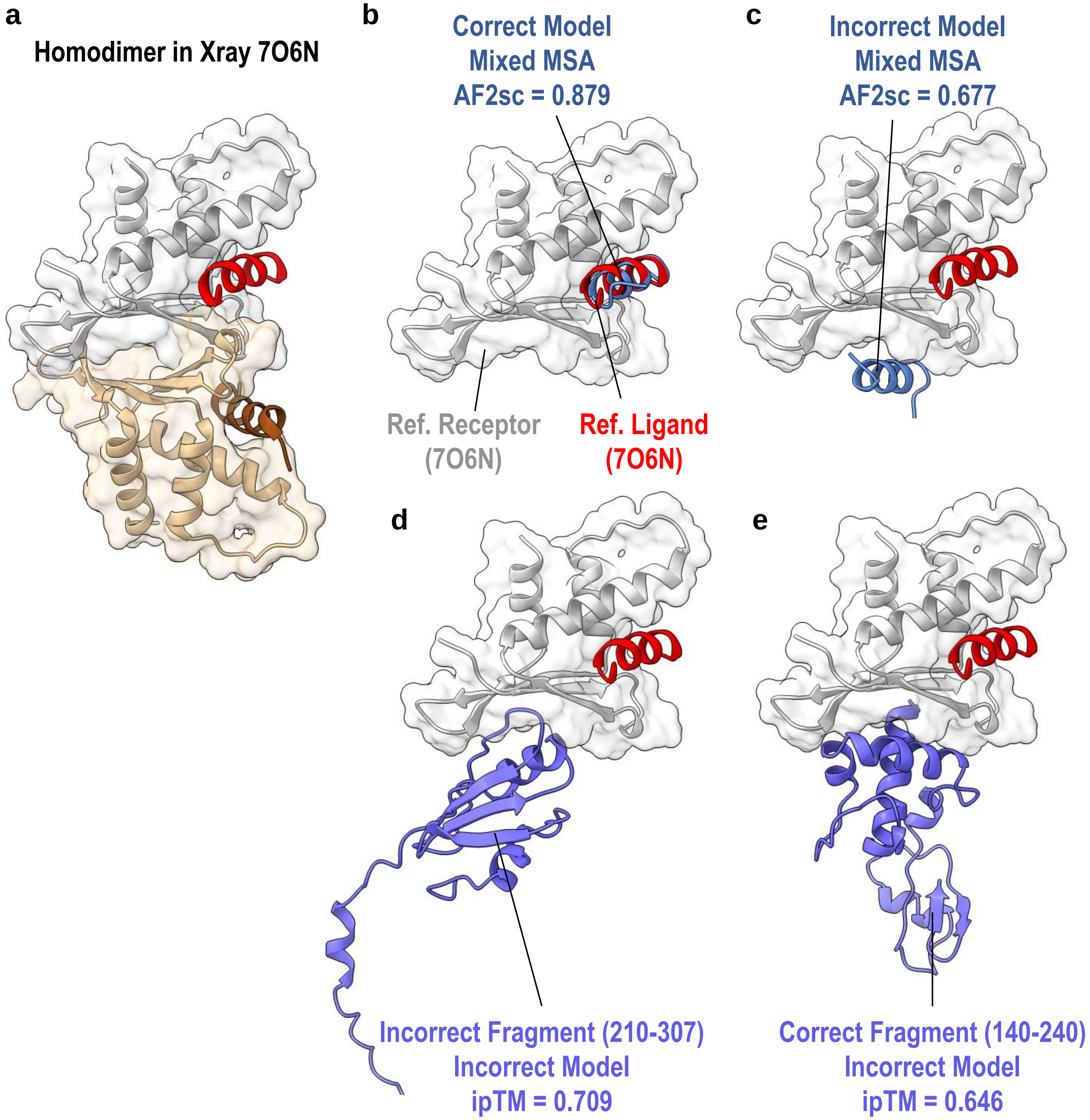

Supplementary Figure 5. Analysis of the 7O6N test case, failures and success.
Focus on the test case PDB:7O6N which was not properly predicted when scanning fragments of the ligand protein against the receptor but was correctly predicted when using delimited peptide. **(a)** Surface representation of the reference X-ray structure of the receptor of 7O6N folding as a homodimer (grey and wheat surface color) and bound to its ligand represented as a red and brown cartoon. **(b)** Model obtained using the mixed-delim-delim protocol with the highest AF2 confidence score superimposed on the reference structure (as in panel a) showing a correct prediction. **(c)** Model obtained using the mixed-delim-delim protocol with a moderate AF2 confidence score binding incorrectly to the receptor domain in the surface involved in the formation of the homodimer. **(d)** Model obtained using the ligand fragment with delimitation 210-307 during the scanning protocol which obtained the highest moderate ipTM score of 0.709 (Figure 4c). **(e)** Model obtained using the ligand fragment with delimitation 140-240 during the scanning protocol which obtained the second highest ipTM score of 0.646 and which overlaps with the correct binding site (Figure 4c). Here, the two fragments were incorrectly predicted as they bound a surface normally involved in the formation of the homodimer. Without modeling the receptor as a homodimer, AF2 is misled regarding the location of the proper binding site as the ligand size is increased.

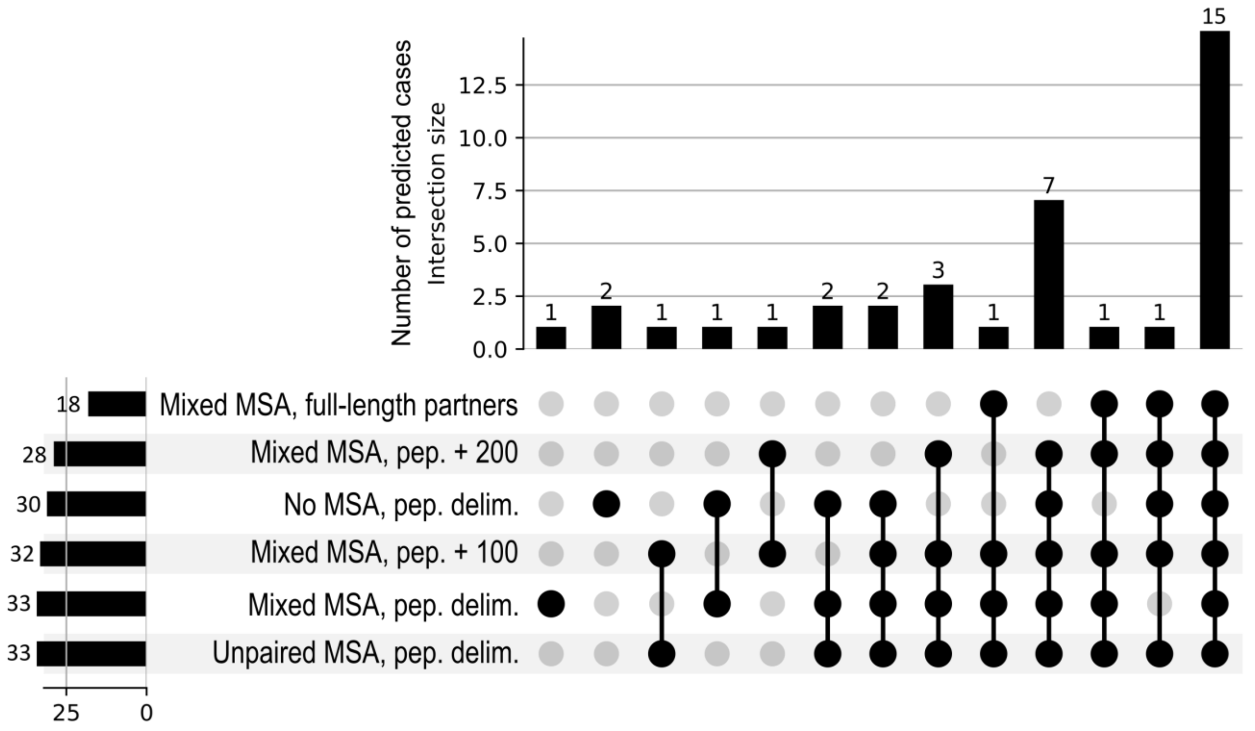

Supplementary Figure 6. Comparison of successful predictions in different prediction modes. UpSet diagram as in Figure 4a for different prediction modes: full-length with mixed alignment, delimited receptor with a ligand extended by a fragment of size 200 with mixed alignment, delimited receptor and peptide without MSA for the peptide, delimited receptor with a ligand extended by a fragment of size 100 with mixed alignment, delimited receptor and peptide with mixed alignment and delimited receptor and peptide with unpaired alignment.

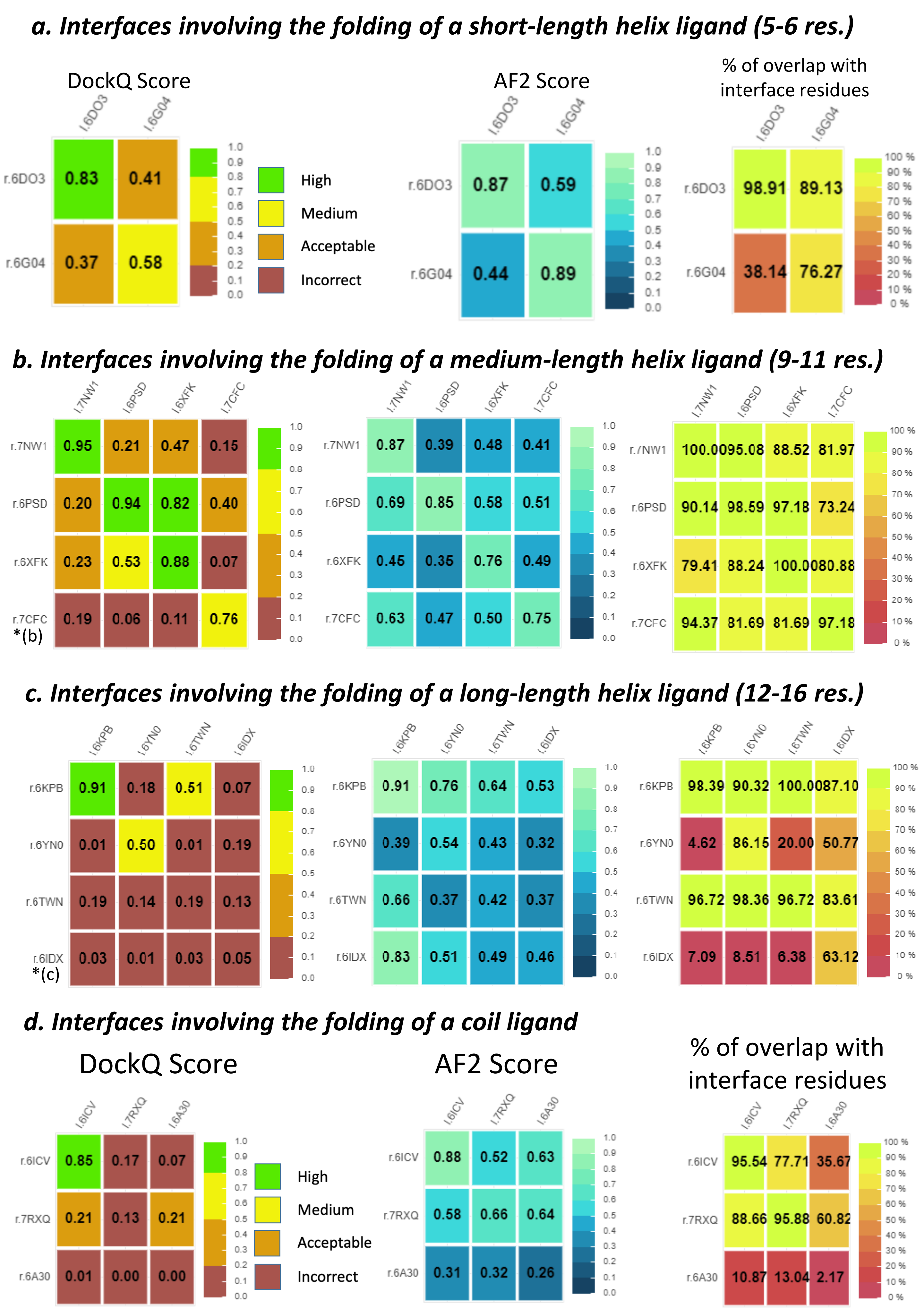

a. Interfaces involving the folding of a short-length helix ligand (5-6 res.)
DockQ Score
AF2 Score
% of overlap with interface residues
l.6DO3 l.6G04
r.6DO3 0.83 0.41
r.6G04 0.37 0.58
High
Medium
Acceptable
Incorrect
r.6DO3 0.87 0.59
r.6G04 0.44 0.89
r.6DO3 98.91 89.13
r.6G04 38.14 76.27
b. Interfaces involving the folding of a medium-length helix ligand (9-11 res.)
l.7NW1 l.6PSD l.6XFK l.7CFC
r.7NW1 0.95 0.21 0.47 0.15
r.6PSD 0.20 0.94 0.82 0.40
r.6XFK 0.23 0.53 0.88 0.07
r.7CFC 0.19 0.06 0.11 0.76
*(b)
r.7NW1 0.87 0.39 0.48 0.41
r.6PSD 0.69 0.85 0.58 0.51
r.6XFK 0.45 0.35 0.76 0.49
r.7CFC 0.63 0.47 0.50 0.75
r.7NW1 100.00 95.08 88.52 81.97
r.6PSD 90.14 98.59 97.18 73.24
r.6XFK 79.41 88.24 100.00 80.88
r.7CFC 94.37 81.69 81.69 97.18
c. Interfaces involving the folding of a long-length helix ligand (12-16 res.)
l.6KPB l.6YN0 l.6TWN l.6IDX
r.6KPB 0.91 0.18 0.51 0.07
r.6YN0 0.01 0.50 0.01 0.19
r.6TWN 0.19 0.14 0.19 0.13
r.6IDX 0.03 0.01 0.03 0.05
*(c)
r.6KPB 0.91 0.76 0.64 0.53
r.6YN0 0.39 0.54 0.43 0.32
r.6TWN 0.66 0.37 0.42 0.37
r.6IDX 0.83 0.51 0.49 0.46
r.6KPB 98.39 90.32 100.00 87.10
r.6YN0 4.62 86.15 20.00 50.77
r.6TWN 96.72 98.36 96.72 83.61
r.6IDX 7.09 8.51 6.38 63.12
d. Interfaces involving the folding of a coil ligand
DockQ Score
AF2 Score
% of overlap with interface residues
l.6ICV l.7RXQ l.6A30
r.6ICV 0.85 0.17 0.07
r.7RXQ 0.21 0.13 0.21
r.6A30 0.01 0.00 0.00
High
Medium
Acceptable
Incorrect
r.6ICV 0.88 0.52 0.63
r.7RXQ 0.58 0.66 0.64
r.6A30 0.31 0.32 0.26
r.6ICV 95.54 77.71 35.67
r.7RXQ 88.66 95.88 60.82
r.6A30 10.87 13.04 2.17

e. Interfaces involving the folding of a helix + strand ligand

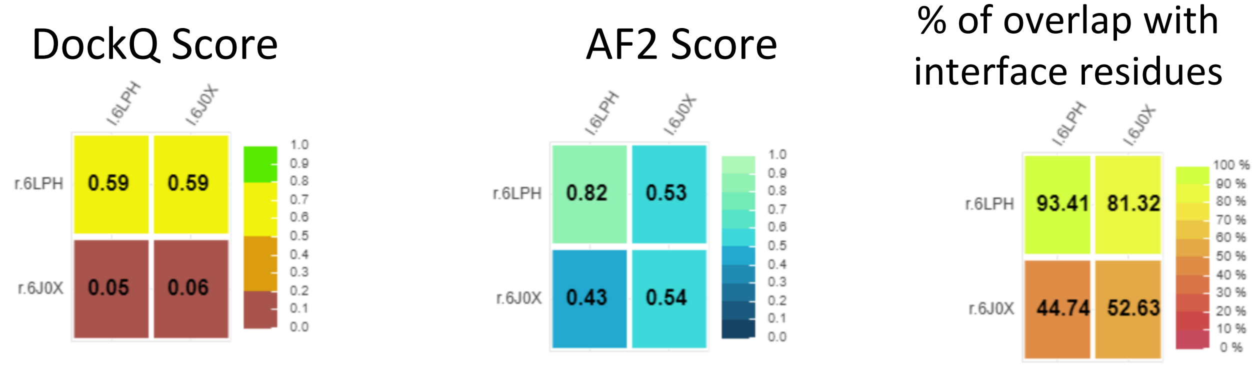

f. Interfaces involving the folding of a single strand ligand (2-3 res.)

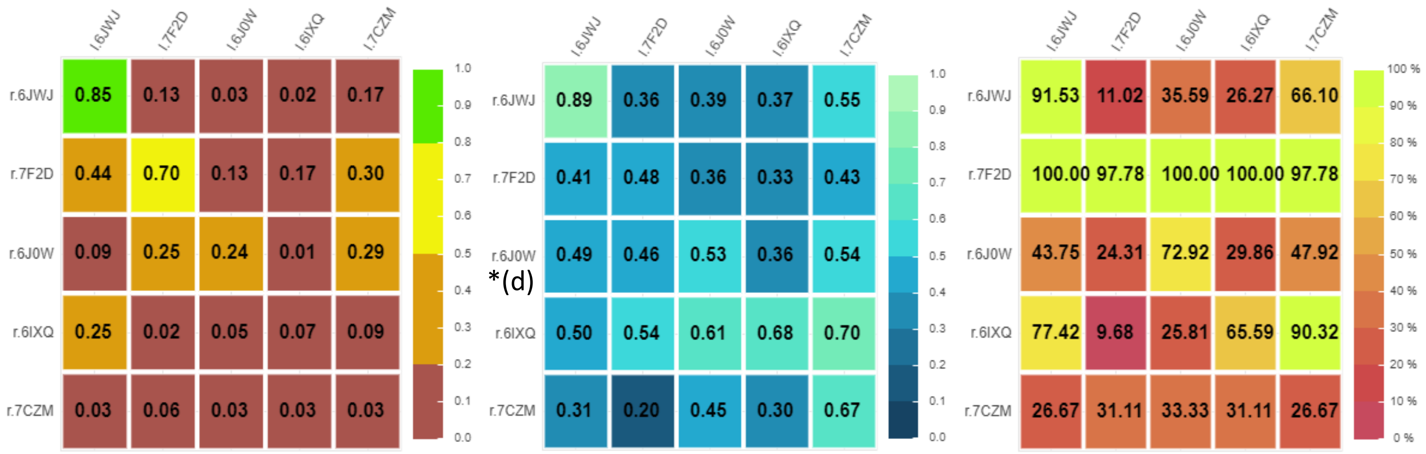

g. Interfaces involving the folding of a two-stranded ligand

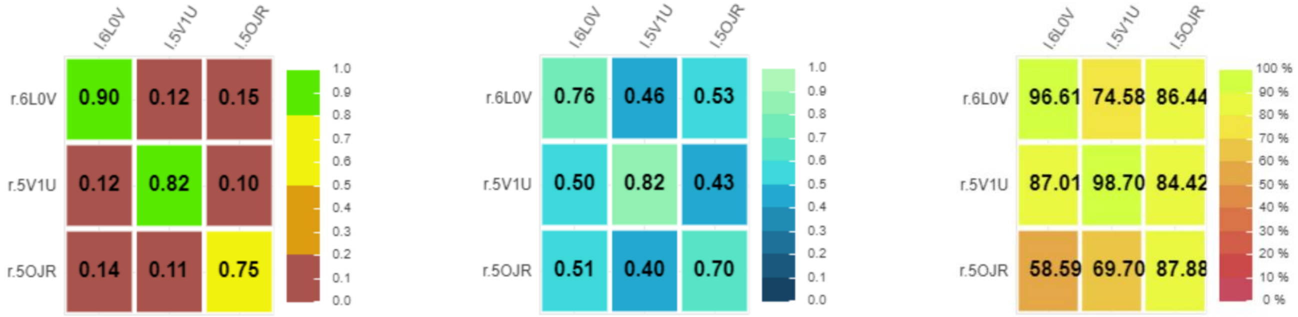

Supplementary Figure 7. Cross-partners AF2 predictions for complexes between receptors and cognate or non cognate peptides from the same cluster.
Cross-partners analyses were performed between cases from the same groups of structures clustered according to the bound ligand conformations: **(a)** short helix, **(b)** medium helix, **(c)** long helix, **(d)** coil, **(e)** helix+strand, **(f)** single strand, **(g)** two strands. For each cross-partner configuration, three matrices are shown: DockQ scores, AF2 combined scores and percentage of overlap with correct interface residues. Matrix rows correspond to the same receptor (labeled as r.PDB) crossed with different peptides while columns report for every ligand (labeled as l.PDB) crossed with different receptors. Color code for DockQ score matrices is brown for Incorrect, orange for Acceptable, yellow for Medium and green for High; for AF2 confidence score matrices, the colors range from dark blue to light green for AF2 scores from 0 to 1; for the fraction of overlap with correct interface residues, the colors range from red to yellow for percentages between 0% to 100%. The cases represented as structural models in the panels (b), (c) and (d) of Figure 5 are labeled on the lines of the corresponding matrices with an asterisk and the corresponding panel letter.

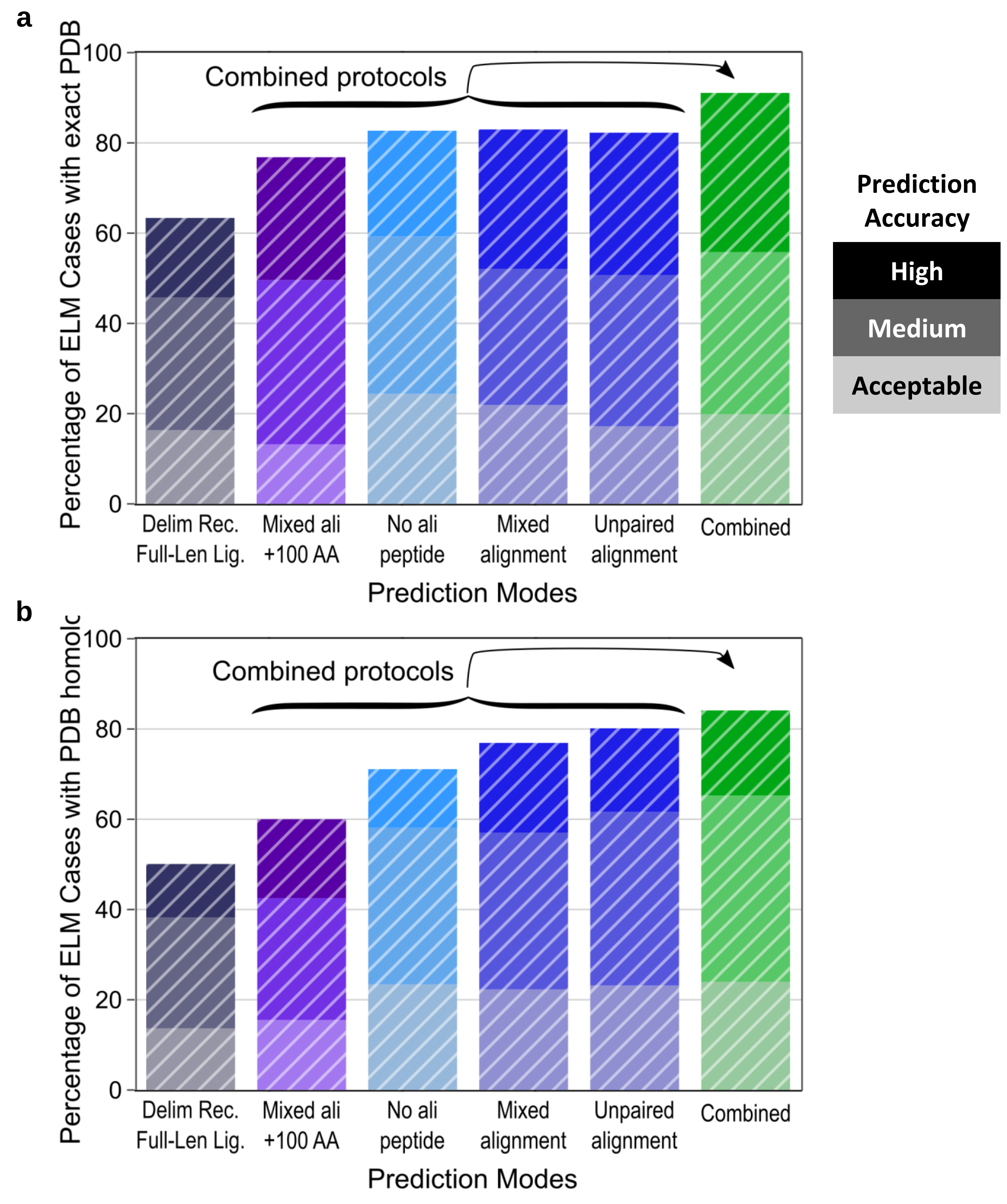

Supplementary Figure 8. Success rates on two subsets of the ELM dataset, with either an identified exact PDB reference or at least one identified homologous PDB reference.
As in Figure 8, the histograms represent the mean success rates obtained from the bootstrap sampling of 1000 iterations over the ELM categories, randomly selecting one ELM complex in each of the categories. At every iteration, success rates were calculated and the mean success rate value is reported in the histogram where the best AF2-Multimer model (best AF2 confidence score) is of Acceptable (light color), Medium (medium color) or High (dark color) quality according to the CAPRI criteria for protein-peptide complexes (see Methods). Six different protocols were assessed and their average success rates reported with same color codes as in Figures 2 and 5. **(a)** The sampling was carried out exclusively among ELM cases for which an exact PDB reference was available. There were 79 ELM categories with at least one exact PDB reference. **(b)** The sampling was carried out exclusively among ELM cases for which a homologous PDB reference was available. There were 73 ELM categories with at least one homologous PDB reference.

Supplementary Table 1: Table of the 42 test cases with their main characteristics

INDEX: Index of the case in benchmark dataset
PDB: Reference of the experimental structure used for validation
CHAIN: Label of the chain used in the reference structure
REC/LIG: Status of the molecule either receptor or ligand. A case can have several receptor in case of homodimers or heterodimers
UNIPROT: Reference sequence Uniprot index used to generate the multiple sequence alignments
START: Index of the first residue modeled in the delimited conditions as defined in the SEQRES PDB parameter
STOP: Index of the last residue modeled in the delimited conditions as defined in the SEQRES PDB parameter
DELIM_LENG Length of the chain in the delimited condition
FULL_LENGT Full length of the protein sequence as defined in Uniprot
SS_TYPE: Secondary structures of ligand in the reference complex. Numbers in brackets report the length of each secondary struct. element

#INDEX	PDB	CHAIN	REC/LIG	UNIPROT	START	STOP	DELIM_LENGTH	FULL_LENGTH	SS_TYPE
1	5NCL	A	receptor	P53894	251	756	506	756	-
1	5NCL	B	receptor	P43563	46	287	242	287	-
1	5NCL	D	ligand	P24276	205	214	10	1250	coil
2	5OJR	A	receptor	Q8DI95	39	347	309	347	-
2	5OJR	E	ligand	Q8DIV4	335	352	18	360	strand (3) + strand (3)
3	5V1U	A	receptor	D1CIZ5	1	88	88	88	-
3	5V1U	E	ligand	D1CIY7	1	20	20	65	strand (2) + strand (6)
4	6A30	A	receptor	Q62768	944	1523	580	1735	-
4	6A30	P	ligand	P63045	87	92	6	116	coil
5	6DO3	A	receptor	Q9Y2U9	1	362	362	406	-
5	6DO3	C	ligand	Q9Y6D0	85	91	7	93	helical (5)
6	6G04	A	receptor	Q06672	1	152	152	205	-
6	6G04	B	ligand	P39938	100	119	20	119	helix (6) + coil
7	6ICV	A	receptor	Q86TU7	1	503	503	594	-
7	6ICV	C	ligand	P60709	66	88	23	375	coil
8	6IDX	A	receptor	Q96JJ3	5	515	511	720	-
8	6IDX	C	ligand	Q3UHD1	1471	1495	25	1582	helix (16)
9	6IXQ	A	receptor	P19524	1152	1574	423	1574	-
9	6IXQ	B	ligand	P32364	615	650	36	656	strand (2)
10	6J0W	A	receptor	P38850	1	513	513	1070	-
10	6J0W	C	ligand	P40026	13	41	29	464	strand (3)
11	6J0X	A	receptor	P38850	1	513	513	1070	-
11	6J0X	E	ligand	Q06164	22	37	16	1454	strand (3) + helix (5)
12	6JLH	A	receptor	Q5XJX1	7	267	261	272	-
12	6JLH	B	ligand	P60880	154	170	17	206	helix (16)
13	6JWJ	A	receptor	P33755	113	580	468	580	-
13	6JWJ	C	ligand	P53044	288	305	18	361	strand (3) + coil
14	6KPB	C	receptor	Q9LPR8	1	482	482	482	-
14	6KPB	B	ligand	Q700D2	367	383	17	503	helix (13)
15	6L0V	A	receptor	F4K0X5	1006	1066	61	1075	-
15	6L0V	B	ligand	Q5XVG3	274	287	14	287	strand (3) + strand (5)
16	6LPH	A	receptor	Q9VG38	1	258	258	468	-
16	6LPH	B	ligand	P23647	363	387	25	805	strand (5) + helix (5)
17	6OCG	A	receptor	Q7L8A9	59	305	247	365	-
17	6OCG	B	ligand	Q8N300	26	51	26	66	helix (25)
18	6PSD	G	receptor	Q9BSW2	47	121	75	731	-
18	6PSD	H	ligand	B3KM42	287	311	25	377	helix (10)
19	6PSE	A	receptor	Q8TD16	1	98	98	824	-
19	6PSE	B	receptor	Q8TD16	1	98	98	824	-
19	6PSE	C	ligand	Q9Y6G9	433	458	26	523	helix (10)
20	6RKO	A	receptor	P0ABJ9	1	522	522	522	-
20	6RKO	H	ligand	A5A618	1	29	29	29	helix (25)
21	6RKO	A	receptor	P0ABJ9	1	522	522	522	-
21	6RKO	X	ligand	P56100	1	37	37	37	helix (24)
22	6SAT	A	receptor	Q8NNN6	64	152	89	152	-
22	6SAT	B	receptor	Q8NNN6	64	152	89	152	-
22	6SAT	P	ligand	P94337	433	442	10	442	helix (5)
23	6TWN	B	receptor	P26039	1359	1659	301	2541	-
23	6TWN	C	ligand	P06493	207	223	17	297	helix (14)
24	6XFK	A	receptor	P0CL43	84	147	64	147	-
24	6XFK	B	ligand	P35672	543	558	16	562	helix (10)
25	6YN0	A	receptor	P02919	58	804	747	844	-
25	6YN0	B	ligand	P29131	75	93	19	319	helix (12)
26	7B1J	A	receptor	Q9Y6D9	597	718	122	718	-
26	7B1J	B	receptor	Q9Y6D9	597	718	122	718	-
26	7B1J	C	ligand	O43683	455	479	25	1085	helix (15)
27	7CFC	A	receptor	A1ZAC4	272	512	241	746	-
27	7CFC	F	ligand	Q7PLK0	63	78	16	867	helix (9)
28	7CZM	A	receptor	Q8TDY2	1490	1594	105	1594	-
28	7CZM	C	ligand	Q96CV9	173	185	13	577	strand (3)
29	7F2D	A	receptor	P93026	20	182	163	623	-
29	7F2D	B	ligand	P15455	468	472	5	472	strand (2)
30	7MKK	B	receptor	Q9W3W6	14	90	77	3313	-
30	7MKK	C	ligand	Q9W2H9	83	109	27	541	helix (18)
31	7MU2	A	receptor	Q9Y4P8	11	363	353	454	-
31	7MU2	B	ligand	E7EVC7	207	230	24	624	helix (19)
32	7NW1	AAA	receptor	Q9Y3C8	1	167	167	167	-
32	7NW1	FFF	ligand	Q9GZZ9	389	404	16	404	helix (11)
33	7QDW	A	receptor	Q8I2Y4	265	332	68	332	-
33	7QDW	B	ligand	Q8IK99	817	841	25	852	helix (18)
34	7RXQ	A	receptor	Q9BR39	1	437	437	696	-
34	7RXQ	B	ligand	P07293	1594	1609	16	1873	coil
35	7SID	A	receptor	Q13315	1	3056	3056	3056	-
35	7SID	B	ligand	O60934	727	754	28	754	coil
36	6J08	A	receptor	Q3KP22-3	2	109	108	176	-
36	6J08	D	ligand	Q8NHR7	174	209	36	220	strand (2) + strand (3) + strand (5)
37	6ZW0	A	receptor	Q57968	2	293	292	293	-
37	6ZW0	C	ligand	Q58261	140	169	30	241	helix (8) + strand (3) + strand (4)
38	7N40	A	receptor	Q09028	1	425	425	425	-
38	7N40	B	receptor	Q5TKA1	98	274	177	542	-
38	7N40	C	ligand	Q96GY3	95	126	32	246	strand (4) + strand (4) + helix (3) + helix (11)
39	7O6N	A	receptor	Q20057	1	99	99	113	-
39	7O6N	D	ligand	O76616	179	193	15	307	helix (14)
40	5OW5	A	receptor	Q8BG40	481	658	178	658	-
40	5OW5	B	receptor	E9PZI6	3	80	78	493	-
40	5OW5	E	ligand	Q80VC9	461	470	10	1252	helix (9)
41	6JMT	A	receptor	Q80XR8	1	360	360	679	-
41	6JMT	L	ligand	Q9ES28	685	705	21	862	helix (14)
42	6GP7	B	receptor	P0CI74	4	64	61	98	-
42	6GP7	C	receptor	P0CI74	4	64	61	98	-
42	6GP7	D	ligand	A0A5D4ND23	1	17	17	908	helix (9)

Supplementary Table 2: Table of the 7 clusters that were used to sample the cross-partner interactions

# Interfaces involving the folding of a short-length helix ligand (5-6 residues)

index	pdb
5	6DO3
6	6G04

# Interfaces involving the folding of a medium-length helix ligand (9-11 residues)

18	6PSD
24	6XFK
27	7CFC
32	7NW1

# Interfaces involving the folding of a long-length helix ligand (12-16 residues)

8	6IDX
14	6KPB
23	6TWN
25	6YN0

# Interfaces involving the folding of a coil ligand

4	6A30
7	6ICV
34	7RXQ

# Interfaces involving the folding of a helix + strand ligand

11	6J0X
16	6LPH

# Interfaces involving the folding of a single strand ligand (2-3 residues)

9	6IXQ
10	6J0W
13	6JWJ
28	7CZM
29	7F2D

# Interfaces involving the folding of a two-stranded ligand

2	5OJR
3	5V1U
15	6L0V
