# Supplementary material for: From interaction networks to interfaces, scanning intrinsically disordered regions using AlphaFold2
Source: Nat Commun. 2024 Jan 18;15:597. doi: 10.1038/s41467-023-44288-7 (PMC10796318; doi:10.1038/s41467-023-44288-7)
Supplement: Supplementary file 9 — Reporting Summary [file 41467_2023_44288_MOESM9_ESM.pdf]

nature portfolio

Corresponding author(s): Jessica Andreani, Raphael Guerois

Last updated by author(s): Nov 30, 2023

Reporting Summary

Nature Portfolio wishes to improve the reproducibility of the work that we publish. This form provides structure for consistency and transparency in reporting. For further information on Nature Portfolio policies, see our Editorial Policies and the Editorial Policy Checklist.

Statistics

For all statistical analyses, confirm that the following items are present in the figure legend, table legend, main text, or Methods section.

n/a	Confirmed	
☐	☒	The exact sample size (*n*) for each experimental group/condition, given as a discrete number and unit of measurement
☒	☐	A statement on whether measurements were taken from distinct samples or whether the same sample was measured repeatedly
☒	☐	The statistical test(s) used AND whether they are one- or two-sided<br>*Only common tests should be described solely by name; describe more complex techniques in the Methods section.*
☒	☐	A description of all covariates tested
☒	☐	A description of any assumptions or corrections, such as tests of normality and adjustment for multiple comparisons
☐	☒	A full description of the statistical parameters including central tendency (e.g. means) or other basic estimates (e.g. regression coefficient) AND variation (e.g. standard deviation) or associated estimates of uncertainty (e.g. confidence intervals)
☒	☐	For null hypothesis testing, the test statistic (e.g. *F*, *t*, *r*) with confidence intervals, effect sizes, degrees of freedom and *P* value noted<br>*Give P values as exact values whenever suitable.*
☒	☐	For Bayesian analysis, information on the choice of priors and Markov chain Monte Carlo settings
☒	☐	For hierarchical and complex designs, identification of the appropriate level for tests and full reporting of outcomes
☐	☒	Estimates of effect sizes (e.g. Cohen's *d*, Pearson's *r*), indicating how they were calculated

Our web collection on statistics for biologists contains articles on many of the points above.

Software and code

Policy information about availability of computer code

Data collection	No specific software was used for data collection.

Data analysis	- Software MMalign (version 20191021, downloaded from https://zhanggroup.org/MM-align/)<br>- Server PPI3D (http://bioinformatics.ibt.lt/ppi3d/, date of PPI3D query August 2022, on the PDB updated July 20, 2022)<br>- Software MMseqs2 (version edb8223d1ea07385ffe63d4f103af0eb12b2058e, from https://github.com/soedinglab/MMseqs2)<br>- Software hhfilter (version 3.3.0, from https://github.com/soedinglab/hh-suite)<br>- Software MAFFT (version 7.475, from https://mafft.cbrc.jp/alignment/software/)<br>- Software PROFIT (version v3.1 from http://www.bioinf.org.uk/software/swreg.html)<br>- Software Singularity (version V3.8.3 from https://github.com/apptainer/singularity/releases/tag/v3.8.3)<br>- Software ColabFold (version 1.3, from https://github.com/sokrypton/ColabFold, commit 5ddfd0bbadbffc5757ee1912107704aec3cd8c04 with correction for the random seed and display of the ipTMscore) for the 42 non-redundant dataset<br>- Software ColabFold (version 1.5.2, from https://github.com/sokrypton/ColabFold, commit 3e99c44eec189ec27f6d120af851adb7ff6aa2a2) for the 923 ELM dataset<br>- Software AlphaFold2-Multimer (version 2.2.0 running with parameters alphafold_params_2022-03-02.tar for the 42 non-redundant dataset and version 2.3.1 for the 923 ELM dataset)<br>- Software chainsaw (https://github.com/judeWells/chainsaw/, commit 1ec2be55b9558a0aa3e17a78293dbba112d73feb)<br>- Software AFsample (https://github.com/bjornwallner/alphafoldv2.2.0, commit 9f76c2adf55403fd80b9079052716857d77a0396)<br>- Software ChimeraX (version: 1.5rc202211240003 (2022-11-24) from https://www.rbvi.ucsf.edu/chimerax/download.html)<br>- Custom code developed for the current work (mainly python scripts) and allowing to reproduce the output of the study are provided in https://github.com/i2bc/SCAN_IDR (https://doi.org/10.5281/zenodo.10213748)

For manuscripts utilizing custom algorithms or software that are central to the research but not yet described in published literature, software must be made available to editors and reviewers. We strongly encourage code deposition in a community repository (e.g. GitHub). See the Nature Portfolio guidelines for submitting code & software for further information.

Data

Policy information about availability of data

All manuscripts must include a data availability statement. This statement should provide the following information, where applicable:

- Accession codes, unique identifiers, or web links for publicly available datasets
- A description of any restrictions on data availability
- For clinical datasets or third party data, please ensure that the statement adheres to our policy

Databases and datasets used in this study:
- Initial list of protein-peptide complexes retrieved from the PDB server databse on April 1, 2022 (https://www.rcsb.org/)
- Full amino-acid sequence were retrieved from the Uniprot database (https://www.uniprot.org/) using uniprot IDs indicated in the PDB mmcif files.
- The uniref30_2103 database (available at https://colabfold.mmseqs.com/) was used to generate the multiple sequence alignments which are all provided in https://doi.org/10.5281/zenodo.7838023
- The ELM database (available at http://elm.eu.org/downloads.html version July 3, 2023) was used to retrieve all the potential pairs of receptor/ligand complexes

Data availability:
- All the accession codes and the delimitations used are provided without any restrictions in Supplementary Table 1
- All the scores calculated for every generated model of the 42 non redundant dataset are provided in Supplementary Table 3
- All the accessions, delimitations and scores calculated for every best model for each 923 cases of the ELM dataset are provided in Supplementary Table 5
- All the sequence alignments, the calculated models and the reference structures used are provided in: https://doi.org/10.5281/zenodo.7838023

Research involving human participants, their data, or biological material

Policy information about studies with human participants or human data. See also policy information about sex, gender (identity/presentation), and sexual orientation and race, ethnicity and racism.

Reporting on sex and gender	There is no sex- and gender-based analysis in our study since these characteristics are unapplicable to the biological macromolecules that were analyzed in this study.
Reporting on race, ethnicity, or other socially relevant groupings	There is no race, ethnicity, or other socially relevant groupings in our study since these characteristics are unapplicable to the biological macromolecules that were analyzed in this study.
Population characteristics	See above
Recruitment	There was no recruitment in the present study.
Ethics oversight	There was no need for an organization to control these aspects absent from our study.

Note that full information on the approval of the study protocol must also be provided in the manuscript.

Field-specific reporting

Please select the one below that is the best fit for your research. If you are not sure, read the appropriate sections before making your selection.

[x] Life sciences [ ] Behavioural & social sciences [ ] Ecological, evolutionary & environmental sciences

For a reference copy of the document with all sections, see nature.com/documents/nr-reporting-summary-flat.pdf

Life sciences study design

All studies must disclose on these points even when the disclosure is negative.

Sample size	The sample size was constrained by our concern that none of the case analyzed in the study should be similar or redundant with any of the structures that were used for the training of AlphaFold2 parameters. Given the conditions that applied both at the sequence and structural level, the sample size was 42 complexes involving a receptor and a small intrinsically disordered protein ligand.<br>We subsequently expanded the dataset tested with 923 test cases taken from the ELM database with the risk that some of the tested cases have been used during the training of AlphaFold2 parameters.
Data exclusions	The exclusion criteria were predefined prior to any generation and evaluation of structural models.<br>- A first criteria applied to the released date of the PDB structure to ensure no overlap with that of the AlphaFold2 training dataset (keep only PDB structures with released date after May 1st, 2018).<br>- A second criteria described in the Methods section was to exclude any case of complex with significant sequence similarity to protein assemblies present in the PDB database before May 1st, 2018.<br>- A third criteria described in the Methods section was to exclude any case of complex with significant structural similarity to protein assemblies present in the PDB database before May 1st, 2018.<br><br>For the ELM database, we excluded the complexes not supported by a pubmed ID, those which had no PDB reference reported either exact of homologous, thos for which multiple ELM motifs of the same ELM type where present in the ligand side.
Replication	All the structural models were generated from the repetition of 5 independent runs of the AlphaFold2 algorithm with 3 recycles each generating 5 structural models following the recommendation of the AlphaFold2 developers. In most cases the success rates for the production of correct models were consistent among the five replicates. Exceptions to that trend are due to the stochasticity of the AlphaFold2 search and are reported in Supplementary Table 3 reporting all the scores and evaluation grades of all the models sampled for each sample case. A different random seed is used to generate each of the the 5 independent runs of AlphaFold2 and all seeds used are provided in the log of the runs provided in the archive https://doi.org/10.5281/zenodo.7838024 for the 42 non redundant dataset.
Randomization	Not applicable for the 42 non redundant dataset since it was not divided into subgroups . For the ELM dataset, to correct for the unbalanced distribution of ELM motifs within the 84 categories of ELM types, we evaluated the predicted success rates by repeated stratified sampling with 1000 repeats of randomly selecting one ELM motif from each of the 84 ELM type categories.
Blinding	Blinding was not required in the study since the test dataset was defined so that it is not overlapping with any of the structures of complex used to train AlphaFold2 parameters.

Reporting for specific materials, systems and methods

We require information from authors about some types of materials, experimental systems and methods used in many studies. Here, indicate whether each material, system or method listed is relevant to your study. If you are not sure if a list item applies to your research, read the appropriate section before selecting a response.

Materials & experimental systems

n/a	Involved in the study	
☒	☐	Antibodies
☒	☐	Eukaryotic cell lines
☒	☐	Palaeontology and archaeology
☒	☐	Animals and other organisms
☒	☐	Clinical data
☒	☐	Dual use research of concern
☒	☐	Plants

Methods

n/a	Involved in the study	
☒	☐	ChIP-seq
☒	☐	Flow cytometry
☒	☐	MRI-based neuroimaging
